# Unsupervised Domain Adaptation with Shape Constraint and Triple Attention for Joint Optic Disc and Cup Segmentation

**DOI:** 10.3390/s22228748

**Published:** 2022-11-12

**Authors:** Fengming Zhang, Shuiwang Li, Jianzhi Deng

**Affiliations:** Guangxi Key Laboratory of Embedded Technology and Intelligent Information Processing, College of Information Science and Engineering, Guilin University of Technology, Guilin 541006, China

**Keywords:** unsupervised, shape constraint, attention

## Abstract

Currently, glaucoma has become an important cause of blindness. At present, although glaucoma cannot be cured, early treatment can prevent it from getting worse. A reliable way to detect glaucoma is to segment the optic disc and cup and then measure the cup-to-disc ratio (CDR). Many deep neural network models have been developed to autonomously segment the optic disc and the optic cup to help in diagnosis. However, their performance degrades when subjected to domain shift. While many domain-adaptation methods have been exploited to address this problem, they are apt to produce malformed segmentation results. In this study, it is suggested that the segmentation network be adjusted using a constrained formulation that embeds prior knowledge about the shape of the segmentation areas that is domain-invariant. Based on IOSUDA (i.e., Input and Output Space Unsupervised Domain Adaptation), a novel unsupervised joint optic cup-to-disc segmentation framework with shape constraints is proposed, called SCUDA (short for Shape-Constrained Unsupervised Domain Adaptation). A shape constrained loss function is novelly proposed in this paper which utilizes domain-invariant prior knowledge concerning the segmentation region of the joint optic cup–optical disc of fundus images to constrain the segmentation result during network training. In addition, a convolutional triple attention module is designed to improve the segmentation network, which captures cross-dimensional interactions and provides a rich feature representation to improve the segmentation accuracy. Experiments on the RIM-ONE_r3 and Drishti-GS datasets demonstrate that the algorithm outperforms existing approaches for segmenting optic discs and cups.

## 1. Introduction

**Figure 1:**
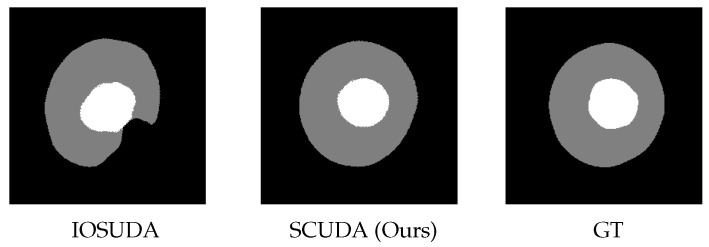
Comparison of the segmentation results between the state-of-the-art method IOSUDA [16] and our SCUDA method on a fundus image. The abbreviation GT refers to ground truth.

Glaucoma is the second most common blinding disease after cataracts [1]. Using fundus pictures, the ratio of the vertical height of the optic cup to the optical disc can be used to determine an early diagnosis of glaucoma. Therefore, it has become a hot topic of research to accurately delineate the optic cup from the optic disc in fundus images and to accurately perform the CDR calculation. At present, deep learning-based techniques for segmenting the optic cup-optical disc have been proven to be effective and have attracted increasing attention in the field. Sevastopolsky et al. [2], for example, proposed a U-Net deep learning network-based method for segmenting the optic cup-optical disc by minimizing the number of convolutional kernels and network complexity. Fu et al. [3] proposed converting the Cartesian coordinates of fundus images into polar coordinate form, and used a U-Net neural network with multi-scale inputs and multi-scale outputs to achieve better performance in optic cup–optical disc segmentation. Most optic cup–optical disc segmentation models work best when the distribution of the test set and training set are the same. Nevertheless, these models tend to perform worse when applied to target domains other than the one they were trained on. This problem is known as a domain shift or distributional shift. Domain adaptation is usually utilized to cope with this problem. According to the information considered for the target task, domain adaptation can be divided into three types, namely, unsupervised, semi-supervised, and supervised domain adaptation. Among them, unsupervised domain adaptation is the one we are most concerned with here. A number of unsupervised domain adaptive algorithms have been proposed for the mitigation of domain shifts in biomedical image segmentation [4,5,6]. For instance, studies of source and target domain domains based on common invariance properties [4,5] concentrate on partitioning the input space of the network. In order to ensure that the segmentation network’s output space is invariant and that the segmentation maps of the source and target regions have the same spatial and geometric shape, [6] employed adversarial learning. Chen et al. [7] proposed an unsupervised framework called IOSUDA for the joint segmentation of the optic cup and optical disc. This framework focuses on separating shared features and stylized features for feature alignment, achieving input and output space alignment, and reducing performance degradation. Although these methods have achieved remarkable performance, they are apt to produce malformed segmentation regions, as demonstrated in Figure 1, that are very far from the real shapes of the optic cup and optical disc. Here, we propose to overcome this issue using a formulation with constraints that, based on the shape of the segmentation region, contain domain-invariant prior information for segmentation networks. The intuition behind our work is that shape information is a strong and valuable prior for optic cup and disc segmentation, as geometrically the optic cup or disc is very close to a round shape. The effectiveness of shape constraints has been proven very recently in 3D pancreas segmentation [8], motivating us to make use of it for the task at issue here. As seen in Figure 1, our method is capable of providing more realistic segmentation results with the proposed shape constraint.

On the other hand, the U-Net [9], a very effective but highly underutilized network introduced by Ronneberger et al. in 2015 for medical image segmentation, serves as the segmentation sub-network in IOSUDA. In order to locate and extract invariant features from the dataset, Zhang et al. [10] suggested a transferable attention U-Net model that used two discriminators and an attention module. Zhao et al. [11] added an attention gate between the encoder-decoder of U-Net in order to concentrate more on the target region, resulting in an attention U-Net architecture. These works suggest that attention mechanisms are effective in boosting the performance of U-Net, which inspires us to attempt a more advanced attention approach for further improvement. Recently, the use of channel attention, spatial attention, or both has been suggested in several studies on computer vision problems as a way to enhance the feature representation ability of by convolutional layers in order to enhance the performance of neural networks. For instance, the Squeeze-and-Excitation (SE) module [12] calculates channel attention and improves performance at a fraction of the cost. Moreover, the Convolutional Block Attention Module (CBAM) [13] and the Bottle-neck Attention Module (BAM) [14] both emphasise the combination of spatial attention and channel attention. Both the BAM (i.e., Bottle-neck Attention Module) and CBAM (i.e., Convolutional Block Attention Module) place emphasis on the union of spatial and channel attention. The Convolutional Triple Attention Module [15] is a lightweight yet effective attention mechanism that calculates attention weights by way of capturing interactions of cross dimensions using a three-branch structure. The segmentation performance of the segmentation sub-network U-Net is improved in this paper using a Convolutional Triple Attention Module (CTAM).

The following may be said about this paper’s contributions:

- We propose a novel unsupervised adaptive framework with shape constraint, called SCUDA, for joint segmentation of the optic cup–optical disc in order to address the problem that existing methods are very likely to produce malformed segmentation regions.
- We exploit a convolutional triple attention module to improve the segmentation network, which is able to capture cross-dimensional interactions and provides rich feature representation in order to boost segmentation accuracy.
- We conducted a number of extensive experiments on the RIM-ONE_r3 dataset and the Drishti-GS dataset to demonstrate the performance of our performed SCUDA framework. The experimental findings verify that SCUDA outperforms the other tested model in terms of performance.

The remainder of the paper is structured as follows: we review related work and describe our methodology in Section 2 and Section 3, respectively; experimental findings are discussed in Section 4; and the work is concluded in Section 5.

## 2. Related Work

### 2.1. Unsupervised Domain Adaptation

A fairly common type of transfer learning is domain adaptation, which generally refers to using a model from one domain and apply it another domain that is only subtly different [17]. Unsupervised domain adaptation in classification is generally built on image and feature alignment [18,19,20,21] between source and target domains. For instance, Long et al. [22] proposed a new network architecture, Deep Adaptation Network (DAN), that used an optimal multi-core selection method for average embedding matching and was able to reduce domain differences. Bousmalis et al. [18] considered shared and private representations of each domain. Unsupervised domain adaptive segmentation has been used for many scenarios, including across various medical images. For example, according to Chen et al. [23], the network can be trained using images from the source domain, with the target domain’s image style being the same as that of the source domain. Huo et al. [24] proposed a Synthetic Segmentation Network (SynSegNet) in order to stylize images from the source domain into those from the target domain. Song et al. [25] introduced several assumptions for feature space extraction; based on this, each loss function was derived and optimized. In addition, to compare the feature spaces of the source, target, and output domains with one another, Chen et al. [26] proposed Synergistic Image and Feature Alignment (SIFA).

### 2.2. Optic Cup–Optical Disc Segmentation

Early work in optic cup–optical disc segmentation focused on hand-crafted features [27,28,29], usually implemented first for target region detection [30,31]. Convolutional neural network-based approaches [3,32,33] have significantly improved accuracy and generalizability. A convolutional neural network for segmentation based on lifting trees was designed by Zilly et al. [32]. Fu et al. [33] proposed the Disc-aware Ensemble Network (DENet) for automated glaucoma screening, which integrates data from local optic disc regions with features from global fundus images. A U-Net based M-Net was proposed by Fu et al. [3] to segment the optic disc-cup, with the segmentation issue converted to a multi-label issue. In addition, a number of semi-supervised methods [34,35] have been proposed to alleviate the problem of insufficient truth labels of the original data. However, these models lack generalization in the face of domain shifting. Recently, unsupervised domain adaptation has made a splash in segmentation of optic cup–optical disc cross data sets [7,36,37]. In order to solve instability in adversarial learning, Liu et al. [36] proposed Collaborative Feature Ensembling Adaptive (CFEA), which makes use of adversarial learning for both the network’s output and intermediate representations. Wang et al. [37] proposed Boundary- and Entropy-driven Adversarial Learning (BEAL), which employs two boundary and entropy discriminators to effectively solve the problem of a target domain’s high entropy and fuzzy boundary. For joint segmentation of the optic disc and cup, Chen et al. [7] proposed an IOSUDA framework including feature and output space alignment while simultaneously introducing adversarial learning into the learning process of segmentation networks; shared features of multiple domains are introduced in the input space. In this paper, we propose an unsupervised domain adaptation with a shape constraint for joint optic disc and cup segmentation. The comparison of our method with previous approaches in terms of used dataset, learning method, supervision method, and use of U-Net, GAN, attention mechanism, and prior geometric constraint (or not) is summarized in Table 1.

### 2.3. Attentional Mechanism

In recent years, many researchers have proposed combining attention mechanisms with deep Convolutional Neural Networks (CNNs) to improve large-scale visualization. Double Attention Networks ($A^{2}$-Nets) [38] use a “double attention block” method that counts and propagates information-rich global features from the input image/video over the entire time and space. Global Second Order Pooling Network (GSoP-Net) [39] uses second-order pooling to collect important features from the entire input space and then distributes them to make further layers easier to verify and disseminate. In addition, an innovative NL block combined with an SE block has been proposed by Global-Context Networks (GC-Net) [40] in an effort to more effectively combine contextual representation with channel weighting. Images can be be segmented and classified using attention processes as well. Criss-Cross networks (CCNet) [41] and SPNet [42] have proposed a new cross-attention module that captures rich contextual information on its cross-paths. A pipeline based on two top-down and two bottom-up attention modules has been presented by Xiao et al. [43] for classifying images.

## 3. Our Approach

### 3.1. SCUDA Framework

**Figure 2:**
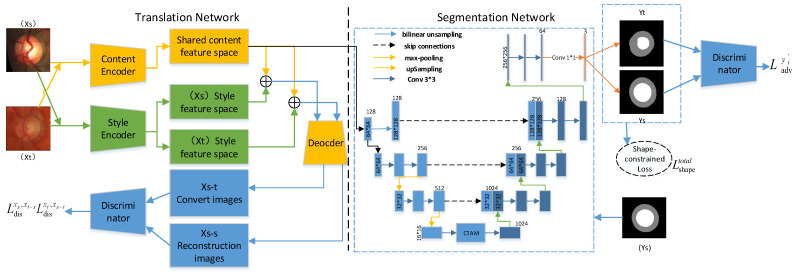
Overview of our proposed SCUDA framework. The left side of the split line is the image translation sub-model and the right side is the image segmentation sub-model; $X_{s-s}$ denotes the reconstructed image dataset and $X_{s-t}$ denotes the converted image dataset, while $Y_{t}$ and $Y_{s}$ denote the segmentation map datasets produced by the splitter network. Additionally, the symbol ⊕ shows the combination of content and style features.

The proposed SCUDA model inherits the IOSUDA pipeline, and is formed from two parts: the image translation model and the segmentation model. Figure 2 shows the overview of SCUDA. The images from the source domain ($X_{s}$), the truth labels from the source domain ($Y_{s}$), and the images from the target domain ($X_{t}$) are the data utilized in training. The picture translation model applies unsupervised transformation between the source and target domains with the goal of learning the shared content features and the corresponding style features. Here, $X_{s-t}$ denotes the transformed image dataset; conversely, $X_{s-s}$ denotes the reconstructed image dataset, while the combination of content and style features is represented by the symbol ⊕. In addition, a shape-constrained loss function $L_{shape}$ for segmentation is designed to incorporate the prior shape information of the segmentation region of the optic cup–optical disc, with the purpose of constraining the segmentation region predicted by the network to ensure that it lies within a feasible configuration space. Moreover, a convolutional triple attention module (CTAM) is adopted for purpose of improving the codec of the segmentation network, which can establish interdependencies between channels or spatial locations to achieve cross-dimensional interactions and boosts segmentation performance. The datasets generated by target-domain and source-domain segmentation are denoted by the variables $Y_{t}$ and $Y_{s}$, respectively. The segmentation network may be optimized via adversarial learning of the segmentation maps of the source and target domains. Additionally, the segmentation maps produced by the target domain are superior.

### 3.2. The Proposed Shape-Constrained Loss Function

**Figure 3:**
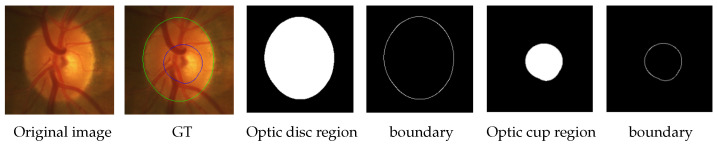
Illustration of the region and boundary of the optic cup and optical disc.

**Figure 4:**
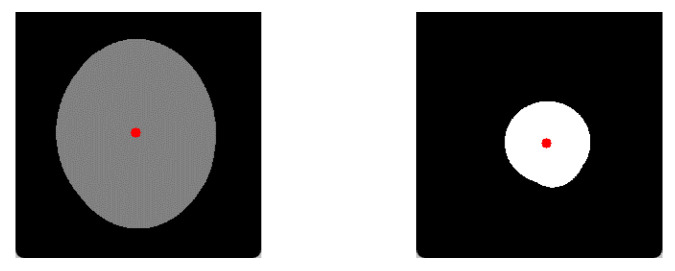
Illustration of the estimated centroids of optic disc (**left**) and optic cup (**right**), which have been marked in color.

Shape information is an important and meaningful a priori indicator for organ segmentation in medical images. Although different datasets of fundus images may appear quite different due to scanning machines, procedures, stages, etc., they should have the same representation of anatomical structures, i.e., contours, of the optic cup and optical disc, which are all circular-like, or at least not very far from a circle. This prior shape information can be used as an indicator to constrain the segmentation results. Specifically, the result of segmentation of a fundus image corresponds to the two contour boundaries of the optic cup and optical disc, respectively, as shown in Figure 3. In the GT diagram in Figure 3, the green contour line indicates the optic disc segmentation and the blue contour line indicates the optic cup segmentation. We denote the set of coordinates of the contour boundaries of the optic cup–optical disc as $I_{cup}$ and $I_{disc}$, respectively. Accordingly, the equation for calculating the center of mass of the optic disc is expressed as

(1) $$\left(C_{X},C_{Y}\right)=C=\frac{1}{k}\sum_{i=1}^{k}m_{i},m_{i}\in I_{disc},$$

where $\left(C_{X},C_{Y}\right)$ denotes the centroid of the optic disc, $C_{X}$, $C_{Y}$ are the x-coordinate and y-coordinate component, respectively. Similarly, the equation for the center of mass of the optic cup is expressed as

(2) $$\left(D_{X},D_{Y}\right)=D=\frac{1}{k}\sum_{i=1}^{k}n_{i},n_{i}\in I_{cup},$$

where $\left(D_{X},D_{Y}\right)$ is the centroid of the apparent cup, $D_{X}$ is the x-coordinate of the center of mass of the apparent cup, and $D_{Y}$ is the y-coordinate of the center of mass of the apparent cup. An illustration of computed centroids is shown in Figure 4, marked by dots.

If the contour boundary of a region is a circle, the distances of each point on the contour to its centroid are equal, and consequently are the same as their mean. In view of this, we use the mean deviation of distances from their mean as a measure of the deviation of a circular-like contour, which is normalized by dividing the mean distance in order to eliminate the scale variations. The proposed shape-constrained loss function for segmenting the optic cup is formulated as follows:

(3) $$L_{cup}=\sqrt{\sum_{i=1}^{k}{\left(E_{cup}^{i}-m_{cup}\right)}^{2}}/m_{cup},$$

where $E_{cup}^{i}={∥C-m_{i}∥}^{2}$, $m_{i}\in I_{cup}$ is the distance of the ith point $\left(i\in \left[1,k\right]\right)$ on the contour of optic cup region to its centroid, k denotes the number of points on the discrete contour, and $m_{cup}$ represents the mean distance, defined by

(4) $$m_{cup}=\frac{\sum_{i=1}^{k}E_{cup}^{i}}{k}.$$

By the same token, we can define the shape-constrained loss function for segmenting the optic disc, which is denoted by $L_{disc}$. Taken together, we obtain the shape constrained loss function $L_{shape}$ for segmenting fundus images as follows:

(5) $$L_{shape}=L_{disc}+L_{cup}.$$

### 3.3. Total Loss Function

The loss function of the SCUDA framework includes the loss of the image translation module and the loss of the image segmentation module in addition to the shape-constrained loss. For the image translation module, let $E_{C}$ denote the content encoder, $E_{S}$ the style encoder, *C* the shared content feature space, $S_{S}$ and $S_{T}$ the style feature space in the source domain and the target domain, respectively, *G* the shared decoder, and L1 the *L*1 distance. For a source domain image $x_{s}\in X_{S}$, $c_{s},c_{t}\in C$, $s_{s}\in S_{S}$, $s_{t}\in S_{T}$, the source domain image loss $L_{rec}^{x_{s}}$, the source domain image content feature loss $L_{rec}^{c_{s}}$, and the source domain image style feature loss $L_{rec}^{s_{s}}$ are defined as follows:

(6) $$L_{rec}^{x_{s}}=E_{x_{s}}{\left[G\left(E_{C}\left(x_{s}\right),E_{S}\left(x_{s}\right)\right)-x_{s}\right]}_{L1},$$

(7) $$L_{rec}^{c_{s}}=E_{c_{s},s_{t}}{\left[E_{C}\left(G\left(c_{s},s_{t}\right)\right)-c_{s}\right]}_{L1},$$

(8) $$L_{rec}^{s_{s}}=E_{c_{t},s_{s}}{\left[E_{S}\left(G\left(c_{t},s_{s}\right)\right)-s_{s}\right]}_{L1},$$

where $E_{z}$ indicates computing the expectation of a function of *z*. The target domain image loss $L_{rec}^{x_{t}}$, its content feature loss $L_{rec}^{c_{t}}$, and its style feature loss $L_{rec}^{s_{t}}$ are defined analogously to the loss of the source domain image. For source domain to target domain image translation, the discriminator $D_{1}$ aims to distinguish the target domain image $x_{t}$ from the transformed image $x_{s-t}$, while the discriminator $D_{2}$ aims to distinguish the source domain image $x_{s}$ from the transformed image $x_{t-s}$, with the former loss function being denoted by $L_{dis}^{x_{t},x_{s-t}}$ and the latter by $L_{dis}^{x_{s},x_{t-s}}$. The $L_{dis}^{x_{t},x_{s-t}}$ loss is formulated as

(9) $$L_{dis}^{x_{t},x_{s-t}}=E_{c_{s},s_{t}}\left[\log_{}\left(1-D_{1}\left(G\left(c_{s},s_{t}\right)\right)\right)\right]+E_{x_{t}}\left[\log_{}D_{1}\left(x_{t}\right)\right].$$

The $L_{dis}^{x_{s},x_{t-s}}$ loss function is defined similarly to $L_{dis}^{x_{t},x_{s-t}}$. The total loss of the image translation model is defined as follows:

(10) $$L_{tra}^{total}=\mu_{1}\left(L_{rec}^{x_{s}}+L_{rec}^{x_{t}}\right)+\mu_{2}\left(L_{rec}^{c_{s}}+L_{rec}^{c_{t}}\right)+\mu_{3}\left(L_{rec}^{s_{s}}+L_{rec}^{s_{t}}\right)+\mu_{4}\left(L_{dis}^{x_{t},x_{s-t}}+L_{dis}^{x_{s},x_{t-s}}\right),$$

where $\mu_{1}$, $\mu_{2}$, $\mu_{3}$, $\mu_{4}$ denote the weights of each component. In the image segmentation module, the segmentation of the optic cup–optical disc is converted to a multi-classification assignment with the segmentation label map $y_{s}\in R^{H\times W\times C}$, where H×W is the image height and width and C is the number of categories. The segmentation network takes $c_{s}$ as input to obtain a predicted segmentation map $y_{s}^{{}^{\prime }}$; similarly, $c_{t}$ is taken as input to obtain a predicted segmentation map $y_{t}^{{}^{\prime }}$. In addition, the role of the discriminator *D* is to determine that $y_{s}^{{}^{\prime }}$ is true and $y_{t}^{{}^{\prime }}$ is spurious. The output size of the discriminator is m×n, and its loss function is defined by

(11) $$L_{dis}^{y_{s}^{{}^{\prime }},y_{t}^{{}^{\prime }}}=\sum_{m,n}^{}\log_{}\left({\left(D\left(y_{s}^{{}^{\prime }}\right)\right)}^{\left(m,n\right)}\right)+\log_{}\left(1-{\left(D\left(y_{t}^{{}^{\prime }}\right)\right)}^{\left(m,n\right)}\right).$$

The split loss function of $y_{s}^{{}^{\prime }}$ is as follows:

(12) $$L_{seg}^{y_{s},y_{s}^{{}^{\prime }}}=-\sum_{h\in H,w\in W}^{}\sum_{c\in C}^{}y_{s}^{\left(h,w,c\right)}\log_{}{y_{s}^{{}^{\prime }}}^{\left(h,w,c\right)}.$$

In order to make $y_{t}^{{}^{\prime }}$ and $y_{s}^{{}^{\prime }}$ have similar definitions, the discriminator is confused in order to judge the patches of $y_{t}^{{}^{\prime }}$ as true. The adverse loss is defined by

(13) $$L_{adv}^{y_{t}^{{}^{\prime }}}=\sum_{m,n}^{}\log_{}{(\left(D\left(y_{t}^{{}^{\prime }}\right)\right)}^{\left(m,n\right)})$$

The total loss of the image segmentation network is defined as follows:

(14) $$L_{seg}^{total}=\delta_{1}\left(L_{seg}^{y_{s},y_{s}^{{}^{\prime }}}\right)+\delta_{2}\left(L_{adv}^{y_{t}^{{}^{\prime }}}\right).$$

where $\delta_{1}$ and $\delta_{2}$ denote the weights of each component. Because of the source and target domains, there are four terms of shape constrained losses during gradient backpropagation. The total shape-constrained loss function $L_{shape}^{total}$ is therefore

(15) $$L_{shape}^{total}=L_{shape}^{x_{s}}+L_{shape}^{x_{t}}+L_{shape}^{x_{s-t}}+L_{shape}^{x_{t-s}}.$$

Taken together, the total loss of the proposed model is

(16) $$L_{total}^{{}^{\prime }}=L_{tra}^{total}+\rho_{1}L_{seg}^{total}+\rho_{2}L_{shape}^{total}.$$

### 3.4. Convolutional Triple Attention Module (CTAM)

**Figure 5:**
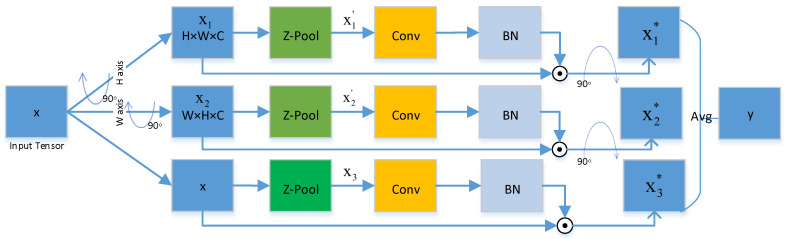
Illustration of the architecture of the convolutional triple attention module (CTAM).

The shared feature content obtained by the image translation model is later fed to the segmentation network for segmentation. Concretely, The segmentation network makes use of an adjusted U-Net, and as the shared content features are downsampled from the original image to be used in the segmentation, the first two downsampling layers of the network are eliminated to satisfy the dimensionality requirement. Convolutional Triple Attention Module (CTAM) [15], a compact yet powerful attention module, is designed and deployed to the interface between the innermost encoder and decoder of U-Net in this paper to further enhance the segmentation network. CTAM captures cross-dimensional interactions of a tensor input by establishing inter-dimensional correlations through a rotation operation and subsequent residual transformations. By computing the attention weights, it generates a large number of feature representations and produces a refined tensor with the same form as the input. The detailed structure of the CTAM is shown in Figure 5. CTAM contains three parallel branches, two each to capture the interaction between channel dimension *C* and a spatial dimension, i.e., *H* or *W*. The output of all three lines is determined using a straightforward averaging method, with one line being utilized to develop spatial attention. More specifically, CTAM accepts an input tensor $x\in R^{C\times H\times W}$, where *C* denotes the number of channels and *H* and *W* denote the height and width of the spatial feature mapping, respectively, which is first passed to each of the three branches. The height and the channel dimension create an interaction in the first branch. Then, *x* is rotated $90^{\circ }$ counterclockwise along with the *H* axis, recorded as $x_{1}$ with the shape $\left(W\times H\times C\right)$, which is minimized to $x_{1}^{{}^{\prime }}$ with the shape $\left(2\times H\times C\right)$ after Z-pool; $x_{1}^{{}^{\prime }}$ later goes through the convolution layer, followed by a batch normalization layer. Moreover, attention weights are obtained by sending them to the sigmoid activation layer. To retain the basic input form of *x*, the created weights are employed in $x_{1}$ and the result is rotated $90^{\circ }$ clockwise along with the *H* axis. The tensor of the first branch that is generated at the conclusion is defined as $x_{1}^{*}$.

Likewise, in the second branch, a $90^{\circ }$ counterclockwise rotation along the *W* axis is applied to *x* with the same principle as in the first branch to obtain the refined $x_{2}^{*}$. The last branch, where the z-pool reduces the channels of the input tensor *x* to two, produces $x_{3}$, which has the shape $\left(2\times H\times W\right)$, and is then processed by a convolution layer. Then, it proceeds successively through a batch normalization layer. Through the sigmoid activation layer, the output generates an attention weight with the shape $\left(1\times H\times W\right)$; the tensor of the final branch generated at the end is defined as $x_{3}^{*}$. The refined tensor of shape $\left(C\times H\times W\right)$ generated by a simple averaging pool of data generated by three branches. To sum up, for an input tensor $x\in R^{C\times H\times W}$, the following equation illustrates how the refinement tensor *y* is obtained from the three branches:

(17) $$y=\frac{x_{1}^{*}\omega_{1}+x_{2}^{*}\omega_{2}+x_{3}^{*}\omega_{3}}{3}.$$

where $\omega_{1}$, $\omega_{2}$, and $\omega_{3}$ are the three cross-dimensional attention weights calculated in the triple attention.

It is worth noting that the incorporation of CTAM into U-Net is based on the following considerations. Despite being widely used, U-Net can be further improved for various segmentation tasks, especially through attention mechanisms, with the motivation of ensuring that the network devotes more focus to the important parts of the data. Remarkably, the parameters related to attention mechanisms can be learned without introducing additional losses. Many works have used attention mechanisms to improve U-Net for medical segmentation [44,45,46,47], including segmentation of the optic disc and cup [10,11,48]. However, the attention methods used in these works require quite a number of learnable parameters, which can easily lead them to suffer from overfiting problems in view of the limited training data in many medical segmentation tasks. Fortunately, a cheap and very effective attention method, namely, CTAM, was proposed in [15] with the aim of capturing cross-dimension interaction while computing attention weights to provide rich feature representations. It has demonstrated the ability to provide similar or better performance to the alternatives. In light of these advantages, in this paper we apply this triplet attention method to boost the performance of U-Net. Because the attention triplet receives an input tensor and outputs a refined tensor of the same shape, it can be applied to any layer to enhance the feature representation there. To avoid increasing too many parameters, we only apply it to the deepest layer of U-Net, as this is the layer with the most abstract representation, which we believe should have the greatest effect on the final result. Trivially, the CTAM becomes an identity map when, say, the convolutional layers in the CTAM have zero kernels and the cross-dimensional attention weights sum to 1. Hopefully, a CTAM can be learned that performs better than this trivial case.

### 3.5. Implementation Details

The network model for this experiment used the Pytorch framework, and training/testing was performed on an RTX3090 with 24 GB of memory. A pre-trained model [3] was used to locate the optic cup and optical disc region of the fundus images in the experimental dataset. Training images were then obtained by cropping and scaling, and the training images were normalized, randomly inverted, and cropped for input. In addition, random seeds were fixed in the experiment. The size of the input training image was 256×256 pixels. The whole model framework was optimized using the Adam method with a batch size of 2 and a training period of 400 epochs, and the initial learning rate was set to $10^{-4}$.

## 4. Experiments

### 4.1. Datasets

**Figure 6:**
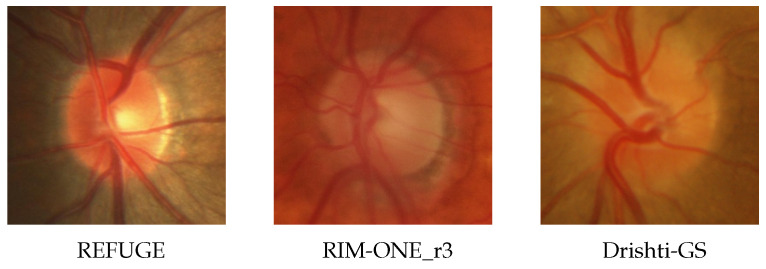
Example fundus images from different datasets. From left to right: REFUGE [50], RIM-ONE_r3 [49], and Drishti-GS [28].

The RIM-ONE_r3 [49] dataset, the Drishti-GS [28] dataset, and the REFUGE [50] dataset were the three publicly available fundus imaging datasets used in this experiment. They have different appearances, as shown in Figure 6. Following [7], the datasets from the source and target domains were split into a training set and a test set for this experiment. The RIM-ONE_r3 dataset with Drishti-GS was employed as the target domain, while the REFUGE training set served as the source domain. Table 2 shows the statistical distribution of the data.

### 4.2. Evaluation Metrics

This experiment uses the IoU coefficient of the optic cup and optic disc along with their Dice coefficient as evaluation indicators. TP (True Positives), FP (False Positives), and TN (True Negatives) are the number of pixels in the segmentation which match the ground truth (for TN/TP) or do not (FP/FN):

(18) $$Dice=\frac{2TP}{FP+2TP+FN},$$

(19) $$IoU=\frac{TP}{FP+TP+FN}.$$

The higher the Dice and IoU values, i.e., the closer they are to 1, the better the segmentation performance of the model. $IoU_{OD}$ and $Dice_{OD}$ denote the IoU and Dice values of the optic disc, respectively, while $IoU_{OC}$ and $Dice_{OC}$ denote the IoU and Dice values of the optic cup, respectively.

### 4.3. Quantitative and Qualitative Analysis

We compare our method with five state-of-the-art methods for segmenting the optic cup–optical disc on two datasets, namely, RIM-ONE_r3 [49] and Drishti-GS [28], to show the efficacy of the SCUDA framework proposed in this study. The methods for comparison are classified into two types. One kind is a model without domain adaptation, such as CycleGAN [51] and Pix2Pix [16]. CycleGAN is an image transformation model based on mismatch, which can transform fundus images into segmentation images to achieve target segmentation. Numerous studies have utilized Pix2Pix, a conditional adversarial generative network (cGAN), for segmentation tasks [52,53]. Another type of unsupervised domain adaptive models include SynSeg-Net [24], SIFA [26], and IOSUDA [7]. In the input space, SynSeg-Net provides picture alignment. Feature alignment and output space alignment are combined by SIFA. Therefore, in our evaluation, CycleGAN and Pix2Pix are trained using the source domain dataset. On the other hand, SynSeg-Net, SIFA, IOSUDA, and the SCUDA model proposed in this paper are trained using data from the source domain and the unlabeled target domain’s training portion, while the test data come from the target domain. Table 3 reports the experimental results. As can be seen, the RIM-ONE_r3 dataset is more difficult than the Drishti-GS dataset, as all the metrics of the tested methods are significantly lower on the former, reflecting the more severe domain drift of the RIM-ONE_r3 dataset compared to the Drishti-GS dataset. Remarkably, our SCUDA method achieves the best performance in terms of all metrics on both datasets. For example, on the RIM-ONE_r3 dataset, our method outperforms the second-best method, IOSUDA, by 1.83%, 2.02%, 1.66%, and 1.73% in $IoU_{OD}$, $IoU_{OC}$, $Dice_{OD}$, and $Dice_{Oc}$, respectively. On the Drishti-GS dataset, our method likewise outperforms the second-best method, again IOSUDA, by 1.26%, 1.70%, 1.41%, and 1.49%, respectively. Results such as those above demonstrate how well our proposed SCUDA model works.

**Figure 7:**
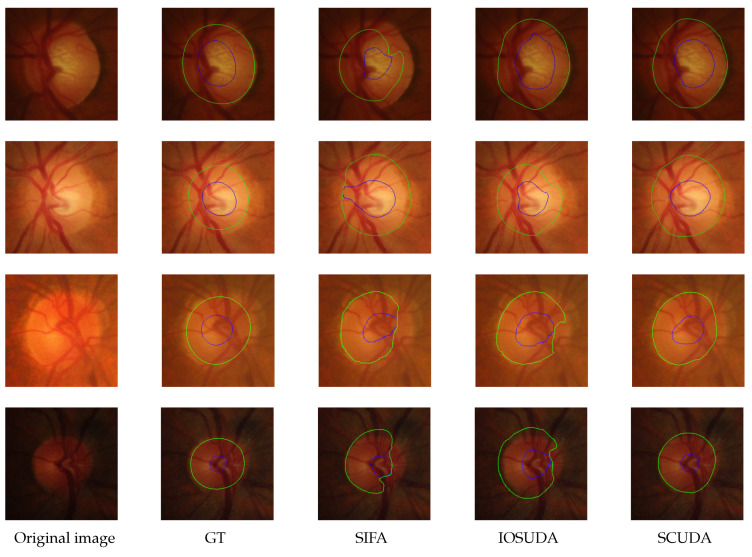
Representative visual examples of the RIM-ONE_r3 test set; the green contours indicate the boundary of the optical disc and the blue contours indicate the boundary of the optic cup. From left to right: original images, GT, and the results of SIFA, IOSUDA, and our proposed SCUDA.

**Figure 8:**
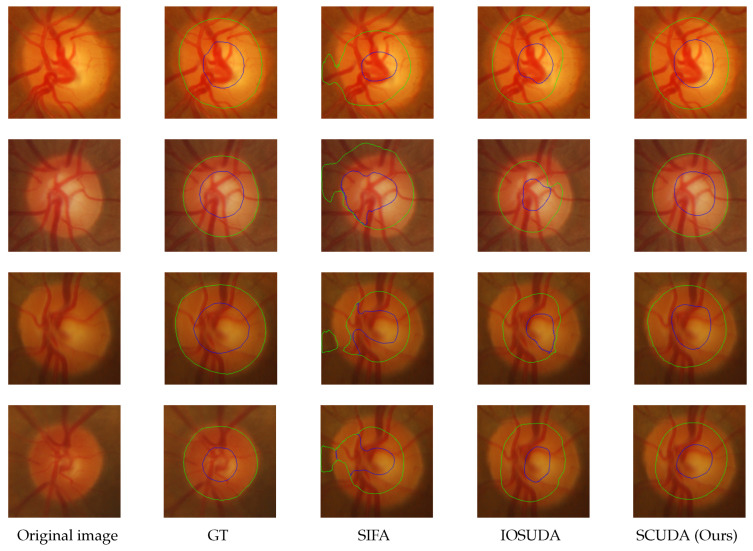
Representative visual examples of the Drishti-GS test set; the green contours indicate the boundary of the optical disc and the blue contours indicate the boundary of the optic cup. From the left to right: original images, GT, and the results of SIFA, IOSUDA, and our proposed SCUDA.

On eight test samples from RIM-ONE_r3 and Drishti-GS, Figure 7 and Figure 8 compare our method qualitatively to two state-of-the-art methods, including the baseline IOSUDA method and SIFA. Concretely, the first and second columns of Figure 7 and Figure 8 show fundus images and the corresponding ground truths, and other columns show the fundus images with the boundary of the optic cup–optical disc marked by different methods. The green contour lines in the figure indicate the optic disc segmentation results and the blue ones indicate the optic cup segmentation results. It can be observed that our SCUDA approach demonstrates better segmentation results with relatively smooth and accurate segmentation contours in all these cases, regardless of whether the fundus images contain clear contours or blur contours, while the other methods produce malformed segmentations in most of these cases. We ascribe this to the effectiveness of the proposed shape constraint, which embeds domain-invariant prior knowledge concerning the circular shape of the optic cup and optical disc into our model.

### 4.4. Ablation Study on the Impact of the Weight of the Shape Constraint

We evaluated the proposed SCUDA on the RIM-ONE_r3 dataset with regard to the weight of the shape constraint loss in order to investigate the effects of the shape constraint weight on the effectiveness of segmentation. The weight ranges were from 0.2 to 2.0 with a step size of 0.2. The four metrics of SCUDA for the different weights are shown in Table 4.

**Figure 9:**
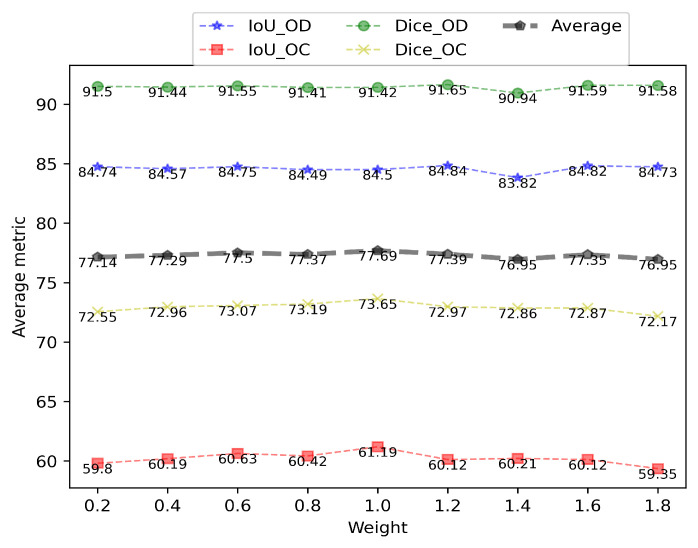
Illustration of trends in the values and means of the four indicators relative to the weights of the shape constraint loss.

**Figure 10:**
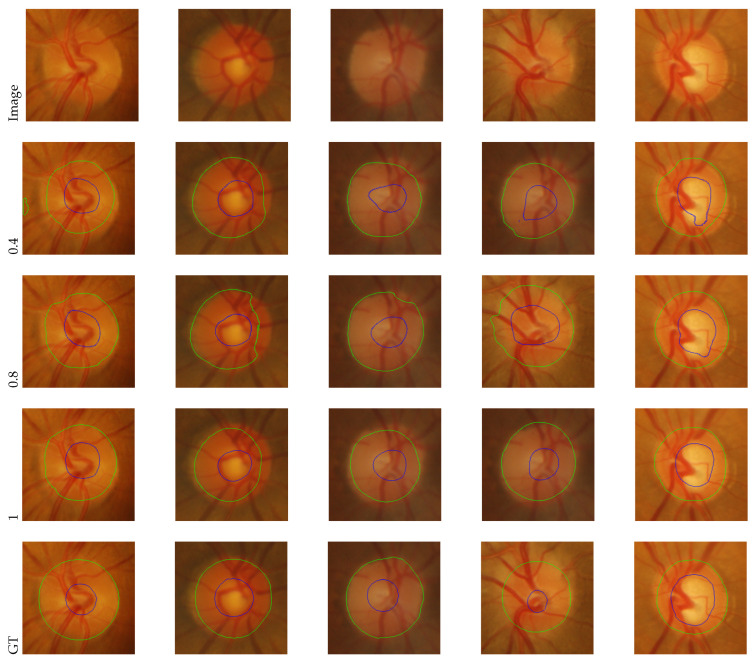
Optic cup–optical disc segmentation with different weights of the loss function.

It can be seen that when the weight of the loss function is 1.2, SCUDA achieves the best $IoU_{OD}$ and $Dice_{OD}$, which are 84.84% and 94.65%, respectively; when the weight is 1.0, SCUDA again achieves the best $IoU_{OC}$ and $Dice_{OC}$, which are 61.19% and 73.65%, respectively. Overall, the best performance is obtained when the weight is 1.0, which is the default setting of the weight in our proposed SCUDA. The trend of the average of the four metrics as the weight changes from 0.2 to 1.8 is plotted in Figure 9 to provide a more intuitive grasp of the influence of this weight. Note that the average of the four metrics is plotted by the gray dotted line. As can be seen, when the weight changes from 0.2 to 1.0, the $IoU_{OC}$ and $Dice_{OC}$ show an increasing trend overall, except for a drop at 0.8. Although the increasing trend is not obvious for IoU_OD and Dice_OD, apparent drops can be observed at 1.4 for both metrics. On average, when the weight increases from 1.0 to 1.8, a decreasing trend is observed on the whole, except for a rise at 1.6. These results suggest that the segmentation performance can be improved if the shape constraint is imposed moderately. In order to understand the impact of the weight of the shape constraint more intuitively, we show five examples of segmentation with different weights in Figure 10. It can be seen that, the segmentation results become visually better and better as the weight goes from 0.4 to 1.0. This justifies the effectiveness of the shape constraint for optic cup–optical disc segmentation and conforms to the fact that the shape-constrained loss function is based on an approximately (though not strictly) correct assumption, namely, that a constraint that is too strong leads to false prior information being imposed on the trained model.

### 4.5. Ablation Study on the Effect of the Proposed Components

**Figure 11:**
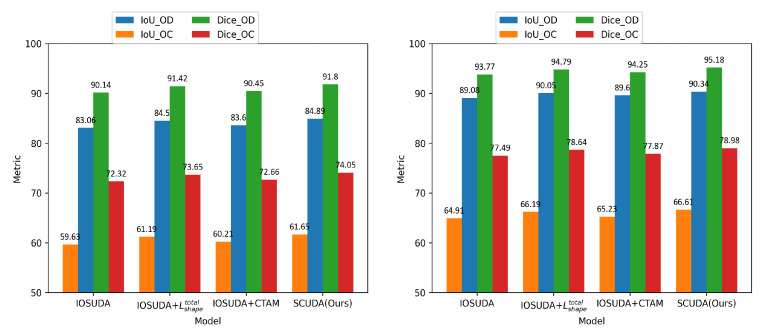
Illustration via bar chart of the effect of the proposed components evaluated on the RIM-ONE_r3 (**left**) and Drishti-GS (**right**) datasets.

To demonstrate the efficiency of the two components proposed in this paper, that is, the shape-constrained loss and the CTAM moduel, an ablation study was carried out. In this experiment, IOSUDA was the baseline model. Depending on whether or not each component was incorporated or not, there were four candidate models: (1) IOSUDA, (2) IOSUDA+ $L_{shape}^{total}$, (3) IOSUDA+CTAM, and (4) SCUDA. IOSUDA+$L_{shape}^{total}$ denotes the addition of the shape-constrained loss function $L_{shape}^{total}$ to the IOSUDA model, IOSUDA+CTAM indicates that the convolutional triple attention module CTAM was added to the IOSUDA model, and SCUDA indicates that the shape-constrained loss function $L_{shape}^{total}$ and the convolutional triple attention module CTAM were both added to the IOSUDA model. These four models were evaluated on the RIM-ONE_r3 and Drishti-GS datasets. Table 5 reports the experimental results. To aid with more intuitive understanding, the results are plotted in the bar chart shown in Figure 11. It can be seen that, compared with IOSUDA, both IOSUDA+$L_{shape}^{total}$ and IOSUDA+CTAM improve the IoU and Dice values of the optic cup and optical disc on the test dataset, which proves the effectiveness of the shape-constrained loss function and the CTAM module proposed in this paper. Specifically, taking $Dice_{OC}$ as an example, IOSUDA+$L_{shape}^{total}$ and IOSUDA+CTAM show improvements of 1.33% and 0.34%, respectively, over the base IOSUDA model on the RIM-ONE_r3 dataset. As for $IoU_{OD}$, on the Drishti-GS dataset, IOSUDA+$L_{shape}^{total}$ and IOSUDA+CTAM show improvements of 0.97% and 0.52%, respectively, over IOSUDA. Overall, the module result of IOSUDA+$L_{shape}^{total}$ is better than the module of IOSUDA+CTAM, although the best outcome on both datasets is only reached when the two modules are combined, that is, in SCUDA. The effectiveness of the proposed components is therefore justified.

## 5. Conclusions

In this paper, we propose an unsupervised domain adaptation with shape constraint for joint optic disc and cup segmentation, which we call SCUDA. A shape-constrained loss function is novelly proposed in this paper, which utilizes domain-invariant prior knowledge about the segmentation region of the optic cup–optical disc in fundus images to constrain the segmentation results during network training. Moreover, we design a convolutional triple attention module in the segmentation network that captures cross-dimensional interactions and provides rich feature representation in order to improve the segmentation performance of the network. Extensive experiments show that the proposed SCUDA framework outperforms state-of-the-art methods for segmentation of the optic cup and optical discs on both the RIM-ONE_r3 and Drishti-GS datasets.

Compared with existing method, we make the first attempt to use prior shape constraints to develop models for joint optic disc and cup segmentation, and use a cheaper yet more effective attention method to boost the performance of U-Net. It is worth noting that, in this work, the shape-constrained loss function is based on an approximate assumption, not a strictly correct one. Our future work will include investigating more realistic shape assumptions to construct constraints for training, along with a more effective and efficient attention mechanism for improving U-Net and novel frameworks of unsupervised domain adaptation for transfer learning.

## Figures and Tables

**Table 1:** Comparison of advantages and disadvantages of our method (SCUDA) with previous methods.

Method	Year	Dataset	Learning Method	Supervisio Method	U-Net	GAN	Attention	Geometric Constraint
[28]	2011	Non-public			×	×	×	×
[27]	2013	SiMES SCES	Traditional learning	Supervised	×	×	×	×
[29]	2013	Non-public			×	×	×	Disc contains cup
[32]	2015	Drishti-GS			×	×	×	×
[3]	2018	ORIGA	Deep learning	Supervised	*√*	×	×	×
[33]	2018	SCES SINDI			*√*	×	×	×
[11]	2021	DRIONS-DB Drishti-GS			*√*	×	*√*	×
[35]	2019	ORIGA REFUGE	Deep learning	Semi- supervised	×	*√*	×	×
[34]	2022	RIGA			*√*	×	×	×
[36]	2019	REFUGE	Deep learning	Unsupervised	*√*	*√*	×	×
[37]	2019	Drishti-GS RIM-ONE-r3 REFUGE			*√*	*√*	×	×
[7]	2021	Drishti-GS RIM-ONE-r3 REFUGE			*√*	*√*	×	×
Ours	2022	Drishti-GS RIM-ONE-r3 REFUGE			*√*	*√*	*√*	Circular-like region

**Table 2:** Statistical distribution of the RIM-ONE_r3, the Drishti-GS, and REFUGE datasets.

Data	RIM-ONE_R3	Drishti-GS	REFUGE
Image size	1072 × 1424	2047 × 1760	2124 × 2056
Quantity of training images	99	50	400
Quantity of test images	60	51	0
Target domain	Target domain	Target domain	Source Domain

**Table 3:** Comparative experimental results of CycleGAN, Pix2Pix, SynSeg-Net, SIFA, IOSUDA, and our proposed SCUDA on the RIM-ONE_r3 test set and the Drishti-GS test set.

Datasets	Model	${\mathrm{IoU}}_{\mathrm{OD}}$ (%)	${\mathrm{IoU}}_{\mathrm{OC}}$ (%)	${\mathrm{Dice}}_{\mathrm{OD}}$ (%)	${\mathrm{Dice}}_{\mathrm{Oc}}$ (%)
RIM-ONE_r3	CycleGAN [51]	70.41	49.76	82.08	64.27
	Pix2Pix [16]	69.57	52.12	81.77	67.81
	SynSeg-Net [24]	71.92	52.69	83.27	67.93
	SIFA [26]	74.67	52.84	84.17	68.03
	IOSUDA [7]	83.06	59.63	90.14	72.32
	SCUDA (Ours)	**84.89**	**61.65**	**91.80**	**74.05**
Drishti-GS	CycleGAN [51]	80.63	45.29	89.12	60.35
	Pix2Pix [16]	82.27	56.02	89.51	69.13
	SynSeg-Net [24]	79.70	49.45	88.36	64.62
	SIFA [26]	83.04	57.29	88.90	70.64
	IOSUDA [7]	89.08	64.91	93.77	77.49
	SCUDA (Ours)	**90.34**	**66.61**	**95.18**	**78.98**

**Table 4:** Illustration of the varied performance of the proposed method on the RIM-ONE_r3 test set with different weights of the shape constraint loss function.

	0.2	0.4	0.6	0.8	1.0	1.2	1.4	1.6	1.8	2.0
$IoU_{OD}$ (%)	84.74	84.57	84.75	84.49	84.50	**84.84**	83.82	84.82	84.73	84.72
$IoU_{OC}$ (%)	59.80	60.19	60.63	60.42	**61.19**	60.12	60.21	60.12	59.35	60.76
$Dice_{OD}$ (%)	91.50	91.44	91.55	91.41	91.42	**91.65**	90.94	91.59	91.58	91.50
$Dice_{OC}$ (%)	72.55	72.96	73.07	73.19	**73.65**	72.97	72.86	72.87	72.17	72.99

**Table 5:** Ablation study on the effect of the proposed components on the RIM-ONE_r3 and Drishti-GS datasets.

Datasets	Model	$L_{\mathrm{shape}}^{\mathrm{total}}$	CTAM	${\mathrm{IoU}}_{\mathrm{OD}}$ (%)	${\mathrm{IoU}}_{\mathrm{OC}}$ (%)	${\mathrm{Dice}}_{\mathrm{OD}}$ (%)	${\mathrm{Dice}}_{\mathrm{OC}}$ (%)
RIM-ONE_r3	IOSUDA [7]	×	×	83.06	59.63	90.14	72.32
	IOSUDA+$L_{shape}^{total}$	*√*	×	84.50	61.19	91.42	73.65
	IOSUDA+CTAM	×	*√*	83.60	60.21	90.45	72.66
	SCUDA (Ours)	*√*	*√*	**84.89**	**61.65**	**91.80**	**74.05**
Drishti-GS	IOSUDA [7]	×	×	89.08	64.91	93.77	77.49
	IOSUDA+$L_{shape}^{total}$	*√*	×	90.05	66.19	94.79	78.64
	IOSUDA+CTAM	×	*√*	89.60	65.23	94.25	77.87
	SCUDA (Ours)	*√*	*√*	**90.34**	**66.61**	**95.18**	**78.98**

## Data Availability

Not applicable.
